# Immunotherapy resistance in non-small-cell lung cancer: From mechanism to clinical strategies

**DOI:** 10.3389/fimmu.2023.1129465

**Published:** 2023-04-06

**Authors:** Suna Zhou, Haihua Yang

**Affiliations:** ^1^ Key Laboratory of Radiation Oncology of Taizhou, Radiation Oncology Institute of Enze Medical Health Academy, Department of Radiation Oncology, Taizhou Hospital Affiliated to Wenzhou Medical University, Taizhou, Zhejiang, China; ^2^ Department of Radiation Oncology, Xi’an No. 3 Hospital, The Affiliated Hospital of Northwest University, Xi’an, Shaanxi, China

**Keywords:** NSCLC, immunotherapy, TME, immune cells, clinical strategies

## Abstract

The high primary resistance incidence and unavoidable secondary resistance are the major clinical obstacle to lasting long-term benefits in Non-small-cell lung cancer (NSCLC) patients treated with immunotherapy. The mechanisms of immunotherapy resistance in NSCLC are complex, mainly involving tumor cells and tumor microenvironment (TME) infiltrating immune cells, including TAMs, B cells, NK cells, and T cells. The selection of clinical strategies for NSCLC progression after immunotherapy resistance should depend on the progressive mode. The progression pattern of NSCLC patients after immunotherapy resistance can be divided into oligo-progression and systemic/multiple progression, which should be considered for further treatment selection. In the future, it needs to explore how to optimize the combined therapy and explore strategies to reprogram infiltrating immune cells under various genetic backgrounds of tumor cells and timely reshape TME during antitumor treatments.

## 1 Introduction

Lung cancer is one of the malignant tumors with the high incidence rate and mortality in the world (1). Non-small-cell lung cancer (NSCLC) accounts for 80% of lung cancer, which mainly includes squamous cell carcinoma (LUSC) and adenocarcinoma (LUAD) (2). The development of immunotherapy profoundly initiates a new era of antitumor treatment, and single or combination immunotherapy has been applicated as the first- and second-line treatment strategies for NSCLC. Inhibitors against programmed death protein 1 (PD-1)/its ligand (PD-L1) or cytotoxic T lymphocyte antigen 4 (CTLA-4) are the most classical and widely applicated immune checkpoint inhibitors (ICIs). Although ICIs profoundly improve overall survival in NSCLC patients with I-IV stages (3–7), there is a non-all-patient response to immunotherapy. Oncologists and NSCLC patients inevitably face the challenge of immunotherapy primary-, secondary-resistance, and progression after treatment discontinuation. As recommended by the first meeting of the SITC Immunotherapy Resistance Taskforce, patients with primary resistance showed that the tumor evaluation after <6 weeks of immunotherapy was progress disease (PD) or stable disease (SD), while secondary resistance was defined as that tumor response to immunotherapy reached complete response (CR), partial response(PR), or SD ≥6 months, and then PD confirmed by imaging scan (8). Explore resolution strategies for improving tumor response to immunotherapy will bring a new leap in NSCLC prognosis. The mechanism of immunotherapy resistance is complex, dynamic, and interdependent. A prerequisite for a clinical response to immunotherapy is a normal cancer-immunity cycle(CIC), which comprises the release of cancer cell antigen, cancer antigen presentation by dendritic cells/APCs, priming and activation of APCs and T cells, trafficking of T cells to tumors, infiltration of T cells into tumors, recognition of cancer cells by T cells, and immune-mediated cancer cells killing (9). ICIs application can block the inhibitory signal of T cell activation, which is only one step of completed CIC. One or more steps of the CIC are interrupted to enable tumors to evade immunosurveillance, and immunotherapy fails to activate effective antitumor immunity (10). During the CIC process, the regulation in the recruitment and infiltration of T cells and cancer cell antigen releasement contribute to the remodeling tumor microenvironment (TME). It also plays a pivotal role in antitumor therapeutic efficacy, except in affecting tumor progression (11). TME is a complex and dynamic changed microenvironment comprising endothelial cells, fibroblasts, immune cells, etc., infiltrating with cytokines, growth factors, hormones, extracellular matrix, etc., and nourishing by the surrounding tumor vascular (11). The TME may dynamically converse between the immunosuppressive TME and the immune-active TME. In addition, the inflammatory status, gut microbiome, diet, etc. of tumor hosts have been demonstrated to be associated with primary and secondary resistance to ICIs (12). The immunological condition of hosts also can affect cells within TME through systemic or local ways (12). Therefore, focusing on the mechanism and drug of TME modulation may be the breakthrough point for reversing immunotherapy resistance. In this review, we focus on the current clinical predicament of immunotherapy resistance in NSCLC, the role of CIC, especially TME, in immunomodulation, and potential strategies to reverse immunotherapy resistance.

## 2 The characteristics of immunotherapy resistance for NSCLC

### 2.1 Primary resistance to immunotherapy

**Figure 1:**
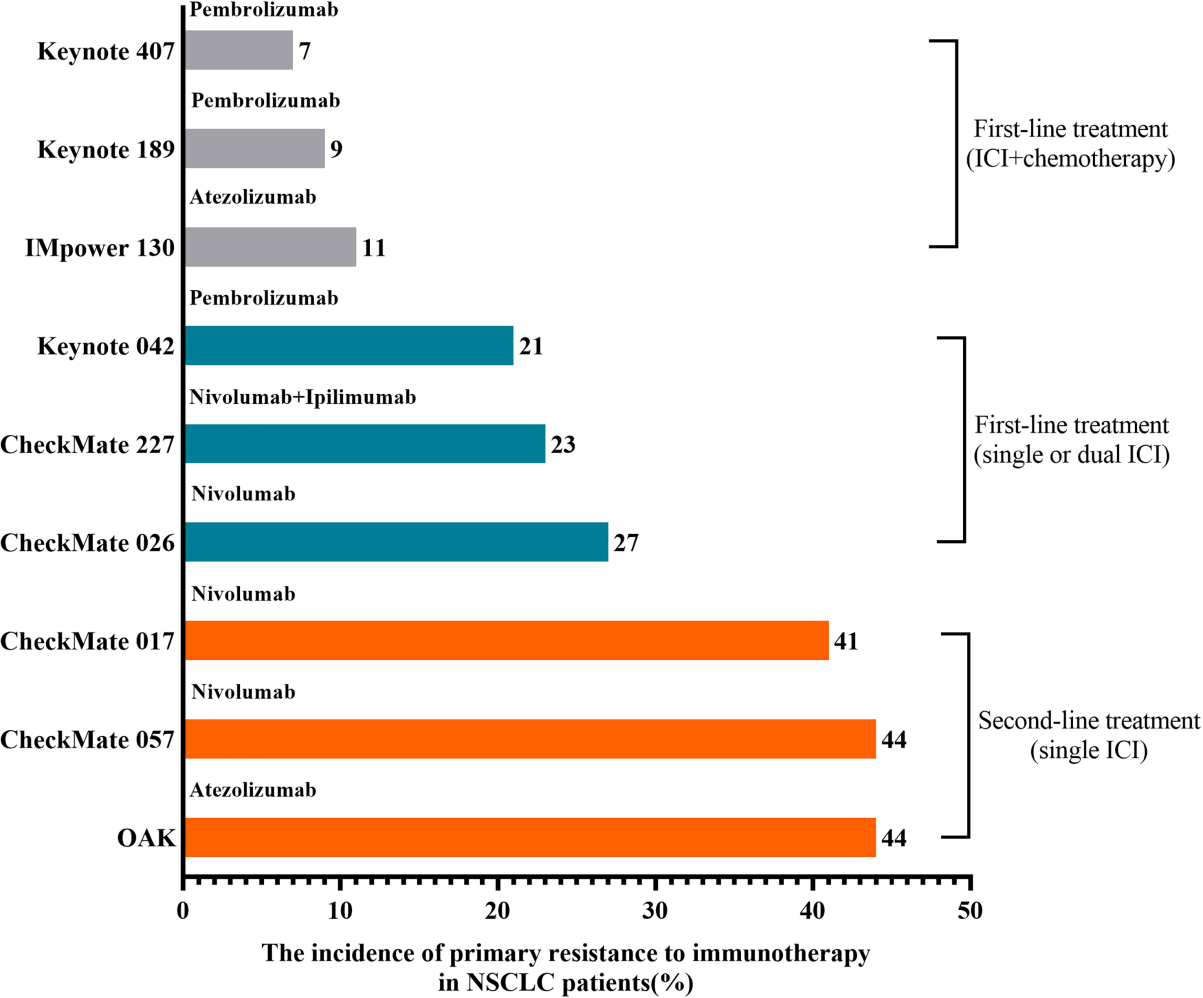
The incidence of primary resistance to immunotherapy in NSCLC patients. The highest incidence of primary resistance to ICIs was found in patients treated with ICIs as second-line treatment after chemotherapy failure, while the lowest incidence was found in NSCLC patients previously treated with immunotherapy plus chemotherapy.

Phase III randomized trials (CheckMate 017 and CheckMate 057) were designed to compare Nivolumab with chemotherapy in the second-line treatment of NSCLC patients with progression after standard first-line treatment (13). The best overall response (BOR) analysis showed that patients with PD accounted for over 40%, and these patients could be defined as primary resistance to ICIs (13). OAK trial also showed that accounting for 44% of NSCLC patients treated with atezolizumab as second-line treatment developed PD (14). For ICI alone or combined with another ICI as first-line treatment, 21%~27% of NSCLC patients have primary resistance to immunotherapy (15–17). For ICI combined with chemotherapy as first-line treatment, the incidence of primary resistance to ICIs in NSCLC patients was around 10% (18–20). As shown in **Figure 1**, the incidence of primary resistance to immunotherapy in NSCLC patients previously treated with standard chemotherapy was higher than that in NSCLC patients treated with ICIs as first-line treatment. Moreover, a lower incidence of primary resistance to ICIs appeared in NSCLC patients treated with immunotherapy plus chemotherapy. This phenomenon may result from the fact that various chemotherapeutic agents can induce immunogenic cell death (ICD) (21). As we know, ICD can enhance the release of cancer cell antigens, a step of a normal cancer-immunity cycle, to improve an anticancer immune response (21, 22).

### 2.2 Secondary resistance to immunotherapy

**Figure 2:**
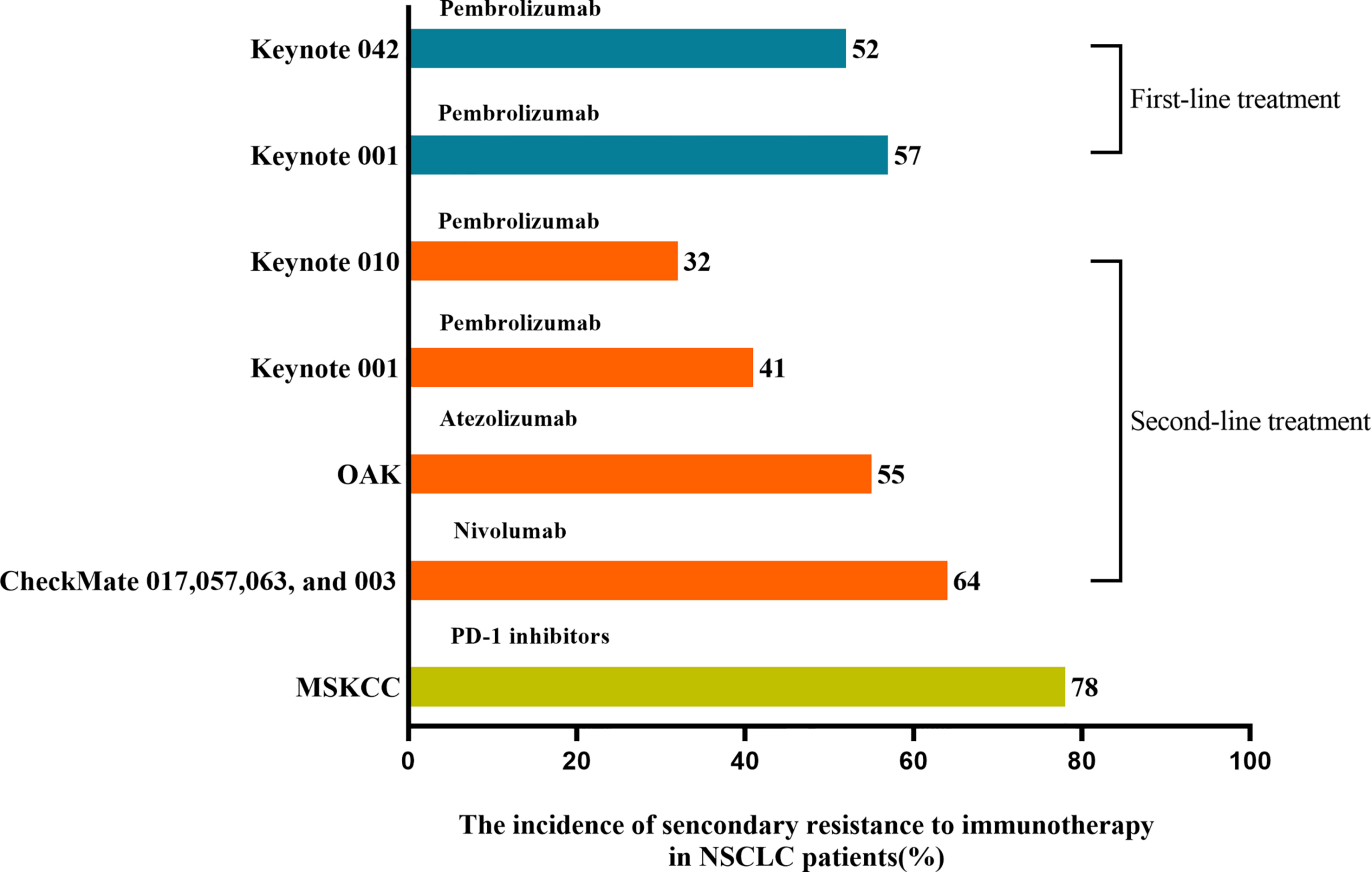
The incidence of secondary resistance to immunotherapy in NSCLC patients. ICIs applied as first- and second-line treatment in treating NSCLC patients resulted in 52%-57% and 32%-64% rates of secondary resistance, respectively. MSKCC found that 78% of patients who acquired an initial response to PD-1 inhibitors further developed secondary resistance.

Acquired resistance was defined as having an initial response to ICIs over a while and ultimately happening to disease progression (23). A pooled analysis from clinical studies in advanced NSCLC patients treated with nivolumab showed that up to 65% of initial responders developed the progressive disease at 4 years of follow-up (24). As shown in **Figure 2**, ICIs applied as first- and second-line treatment for NSCLC patients with rates of secondary resistance was 52%-57% and 32%-64%, respectively (25). In addition, a retrospective study of 1201 NSCLC patients treated with PD-1 inhibitors at Memorial Sloan Kettering Cancer Center (MSKCC) found that 78% of 243 cases acquired an initial response to immunotherapy and further developed secondary resistance (26). With the prolonged response time or progression-free survival (PFS), the occurrence rate of secondary resistance declined with the duration of remission in NSCLC patients treated with ICIs (26). The 1-year incidence rate was 53%, with 37% at 1-2 years and 10% after 2 years (26). Compared to systematic progression, the most common pattern of secondary resistance to ICIs in NSCLC patients was oligo progression, defined as ≤ 2 progressed disease sites (26–28). And NSCLC patients with oligo progression after treating PD-1 inhibitors occurred secondary resistance later than patients with systematic progression and had a better survival prognosis (26). In the second-line and above treatment for NSCLC patients, an earlier or higher objective response rate (ORR) of a single ICI was accompanied by a lower incidence of secondary resistance and more significant long-term survival benefits (24, 25).

**Figure 3:**
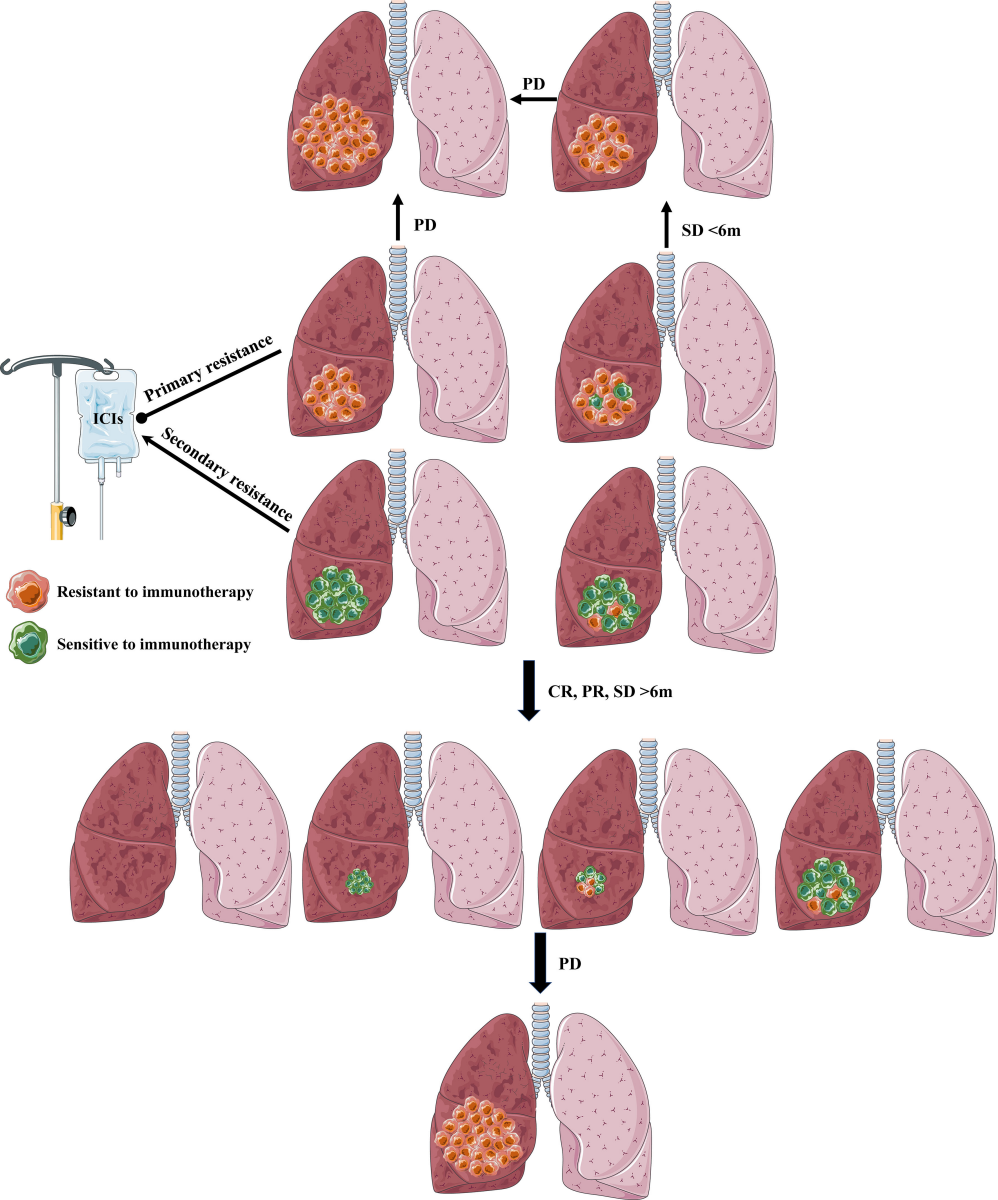
The definition and explanation of primary resistance and secondary resistance. Primary resistance: tumors in primary resistance NSCLC patients may contain no or only a few sensitive tumor cells to immunotherapy, which may present no active immune response (PD) or activate antitumor immune response followed by swiftly submerging by intricated mechanism (SD<6 months). Secondary resistance: When tumors contain no or only a few resistive tumor cells to immunotherapy, NSCLC patients will show a favorable and lasting response (CR, PR, SD) to immunotherapy (>6 months). However, some will form secondary resistance when tumor cells lose active response to immunotherapy by complicated mechanisms.

In summary, tumors in primary resistance NSCLC patients may consist of no or only a few sensitive tumor cells and resistive tumor cells to immunotherapy, which may present no active immune response(PD) or activate antitumor immune response followed by swiftly submerging by intricated mechanism (SD<6 months). When tumors are comprised of no or only a few resistive tumor cells and sensitive tumor cells to immunotherapy, NSCLC patients will show a favorable and lasting response to immunotherapy(CR, PR, SD>6 months). However, some will form secondary resistance when tumor cells lose active response to immunotherapy by complicated mechanisms (**Figure 3**).

## 3 Mechanisms of immunotherapy resistance in NSCLC

**Figure 4:**
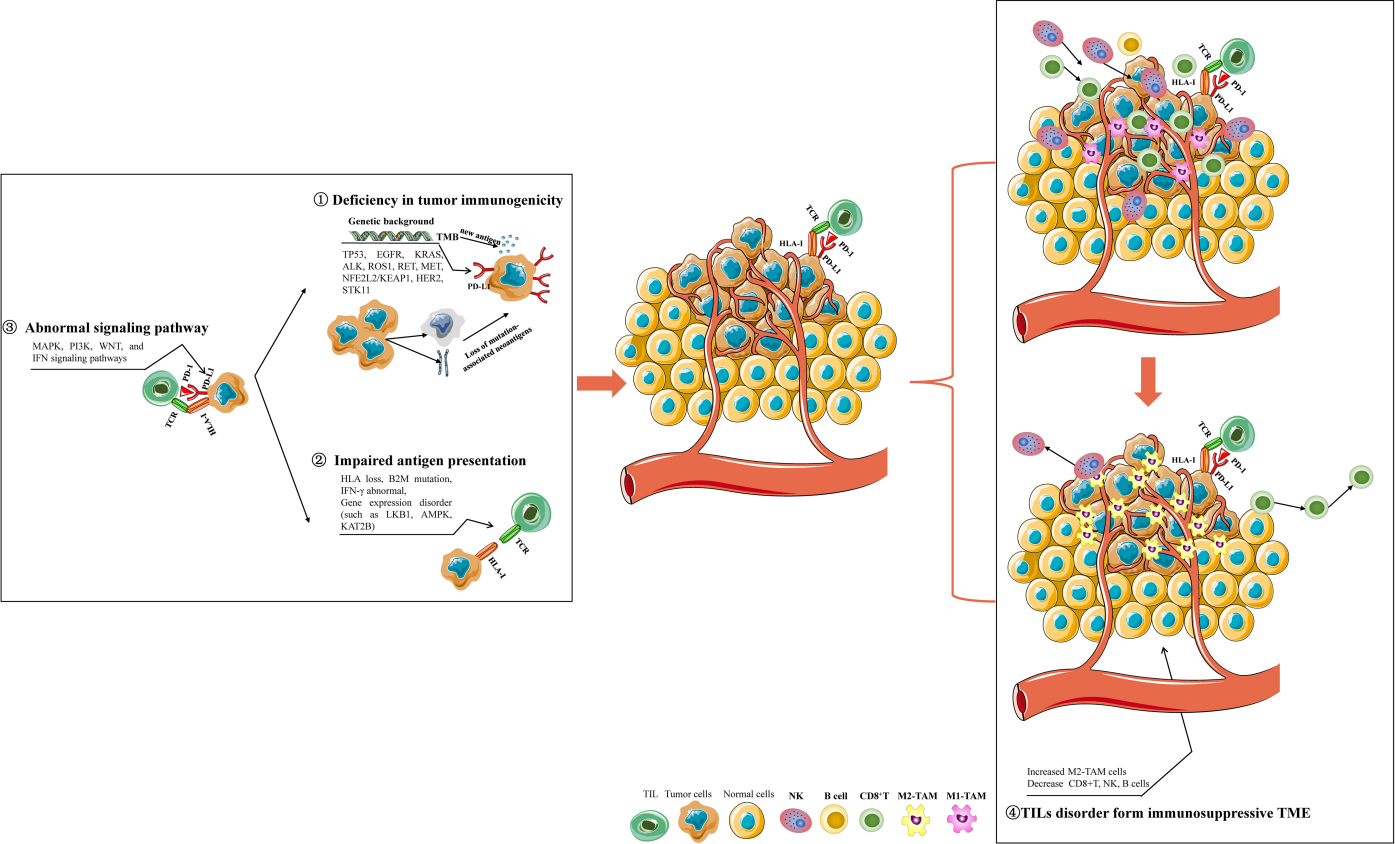
The mechanisms involved in immunotherapy resistance of NSCLC. Deficiency in tumor immunogenicity: various drive gene mutations affect PD-L1 expression, TMB, and tumor-specific neoantigen formation; Impaired antigen presentation: HLA loss, B2M mutation, IFN-γ signaling dysregulation, and some genes disorder; Abnormal signaling pathway: abnormal MAPK, PI3K, WNT, and IFN signaling pathways may influence tumor immunogenicity and antigen presentation; Abnormal activity and infiltration of immune cells in NSCLC TME: NSCLC TME enriched with increased M2 macrophages, decreased B cells, and NK cells form an immunosuppressive TME to resist the initiation of antitumor immunity. Some gene expression disorders or mutations in NSCLC cells can impair the activity, tumor-cell-killing function, proliferation, and infiltration of CD8+ T-cells in TME, which contribute to exhausted CD8+ T-cells for immunotherapy resistance.

A normal CIC, a prerequisite for response to immunotherapy, can be summarized in four steps: recognizable tumor antigen release (within tumor cells); immune cells identify, transmit tumor antigen, and then are activated (system of the tumor-bearing host); recruiting, trafficking, and infiltration of immune cells into the TME (outside of tumor cells); Finally, triggering immune-mediated cancer cell killing(TME, host). A deficit in any one of these four steps will contribute to varying degrees of immunotherapy resistance (**Figure 4**).

### 3.1 Mechanisms involved within NSCLC cells

#### 3.1.1 Deficiency in tumor immunogenicity

PD-L1 expression and tumor mutation burden (TMB) are the most popularly applied biomarkers for predicting immunotherapy response. NSCLC patients with a high expression of PD-L1 usually obtain better benefits from anti-PD-1/PD-L1 therapy (29, 30). NSCLC patients with a higher tumor tissue TMB (tTMB) are correlated with a more effective response to PD-1/PD-L1 inhibitors by leading to tumor-specific neoantigen formation to elevate tumor immunogenicity (31–34). Therefore, any intrinsic and extrinsic factors affecting the expression of PD-L1 or/and TMB in tumor cells may be the mechanism for resistance to immunotherapy. Next-generation sequencing (NGS) applications found that innate drive gene mutations affect PD-L1 expression and TMB, which may contribute to primary or adaptive resistance to immunotherapy in NSCLC. EGFR is a well-studied drive gene in NSCLC patients, the mutation status of which can induce PD-L1 expression (35, 36). In EGFR-mutated NSCLC cells, activated EGFR was found to elevate PD-L1 expression *via* the IL-6/JAK/STAT3 signaling or p-ERK1/2/p-c-Jun pathway (35, 36). Moreover, NSCLC patients with uncommon EGFR mutation, including G719X, L861Q, S768I, and Ex20 ins, had more abundant PD-L1 expression accompanied by CD8^+^ tumor-infiltrating lymphocytes(TILs) infiltration (37). In addition, mutations of RET and HER2 attenuated PD-L1 expression, while mutations of ALK, ROS, and MET enhanced PD-L1 expression but downregulated TMB and TIL infiltration leading to resistance to ICIs (38–41). TP53 or NFE2L2/KEAP1 mutations could increase TMB and PD-L1 expression to enhance the sensibility to immunotherapy (42, 43). However, NSCLC subsets with co-occurrence of EGFR, HER2, ALK, ROS1, RET, and MET mutations get minimal benefit from ICIs despite high PD-L1 expression. These findings confirmed a TMB/PD-L1-independent effect on response sensitivity to ICIs for specific drive genes mutations (44). KRAS mutation, another frequently mutated type in NSCLC, usually co-occurs with different gene mutations. Various KRAS mutation subtypes have distinct TMB and PD-L1 expressions, and KRAS G12C is the most common subtype with a high rate of PD-L1 positive (45). Mutated-TP53 was more prevalent in KRAS wild-type NSCLC, while mutated-STK11 was more frequently found in KRAS-mutated NSCLC (45). In KRAS-mutant lung adenocarcinoma, STK11 mutation was identified as the most common genomic background for primary resistance to PD-1/PD-L1 inhibitors (46). Interestingly, the acquired resistance after initial response to ICIs in NSCLC patients showed the landscape of genomic changes characterized by loss of putative mutation-associated neoantigens through eliminating tumor subclones or deleting specific chromosomal regions (47). Moreover, High TMB and neoantigen burden(APOBEC, IFNGR1, or VTCN1 mutation) were associated with enhanced efficacy response in NSCLC immunotherapy, but PTEN mutation was associated with non-response to immunotherapy (34).

#### 3.1.2 Impaired antigen presentation

Impaired antigen processing and presentation have been confirmed as a mechanism for lung cancer with acquired resistance to ICIs (48, 49). Class I human leukocyte antigen (HLA-I/MHC-I) plays a leading role in neoantigen presentation to improve tumor recognition by T cell receptors. HLA gene loss damages the process of neoantigen presentation resulting in immune evasion for tumors (50). The germline HLA-I evolutionary divergence was strongly associated with the survival benefit of metastatic melanoma or NSCLC patients treated with anti-CTLA-4 or anti-PD-1/-PD-L1 (51). 40% of NSCLC carry allele-Specific HLA Loss(HLA-I LOH), which is related to a raised neoantigen burden, PD-L1 positivity, and poor response to ICIs treatment (52, 53). Loss of the B2M gene, an essential chaperone for HLA-I-mediated antigen presentation, formed an immunosuppressive TME characterized by reduced TIL infiltration and conferred resistance to ICIs in NSCLC (49, 54, 55). Interferon-gamma (IFN-γ) has also been identified to stimulate the expression of HLA on NSCLC cells (56, 57). Except for the role of IFN-γ in HLA regulation, IFN-γ signaling is also critical for the initiation of PD-L1 expression in cancer and host cells (58, 59). An analysis of gene expression profiles for pembrolizumab-treated patients found that IFN-γ-related mRNA profile could predict clinical response to PD-1 inhibitor (60). A prospective study about cytokine profiles in NSCLC patients receiving ICI treatment in the second line revealed that patients with elevated expression of IFN-γ significantly benefit from PD-1 blockers (61). Moreover, attenuated antigen presentation was also found in NSCLC with compromised LKB1 and AMPK activity (62). Low expression of KAT2B concurrent with a higher frequency of somatic genes mutation was associated with lower response efficacy to ICIs in NSCLC patients, while KAT2B was linked to IFN-γ regulation, antigen processing, and presentation (63).

#### 3.1.3 Abnormal signaling pathway

It has been reported that the aberrations of MAPK, PI3K, WNT, and IFN signaling pathways may be implicated in the resistance mechanisms of lung cancer immunotherapy (12). As discussed above, genetic mutation EGFR influences tumor immunogenicity by regulating PD-L1 expression and TMB. MAPK and PI3K pathways were involved in the immunotherapy resistance mechanism mediated by EGFR mutation-induced PD-L1 increasing (64). MAPK and PI3K pathways were downstream of the RAS signaling, the activation of which supported intrinsic PD-L1 expression (65, 66). BRAF mutation is a rare form of NSCLC and is part of the MAPK pathway. And BRAF mutation seems to be more associated with high expression of PD-L1(PD-L1 expression ≥50%) than other subtypes (67). In addition, the activity of the MAPK pathway was significant for EGF- and IFNγ-induced PD-L1 expression, contributing to improving response to ICIs in NSCLC (68). The PI3K/AKT/mTOR pathway plays critical roles in multiple biological functions or processes of cancers, and it can be activated by the genetic mutation of EGFR or KRAS in NSCLC (69–71). Significantly, uncontrolled activation of the PI3K/AKT/mTOR pathway can modulate the response to ICIs by driving PD-L1 expression and remodeling the infiltration and function of TIL (71, 72). WNT signaling alterations in NSCLC were also associated with PD-L1 negativity but this altered PD-L1 expression without predictive value for ICIs (73). However, a recent study identified that SCD1-related fatty acids in serum were correlated with the response efficiency of NSCLC patients treated with a PD-1 inhibitor, while WNT signaling was significantly involved in the immunomodulatory function of SCD1 (74). The loss of IFN signaling in tumor cells has been highlighted as a mechanism of the primary and acquired resistance to ICIs in cancer patients (75–77). The production of IFN in TME could induce PD-L1 expression on the surface of tumor cell lines, including NSCLC (78–80). There is still a lack of evidence about the relation between the genomic alterations in IFN signaling and response to ICIs treatment in NSCLC patients.

### 3.2 Mechanisms involved in NSCLC TME

Recruiting, trafficking and infiltration of immune cells into the TME are essential to a normal CIC. The TME comprised TILs and stromal cells, cytokines, and vasculature, which influence response to immunotherapy by dynamically reshaping the immunogenicity of TME. Immunosuppressive TME formation is a leading mechanism of immunotherapy resistance and is a vital breach to enhance the effective response to immunotherapy. We will focus on the TILs in NSCLC TME to give insights into the immunotherapy resistance mechanisms. A landscape of the TILs in NSCLC TME identified that CD4^+^ T cells were the maximum T cell population, followed by CD8^+^ T cells, and then B cells, macrophages, natural killer (NK) cells, and dendritic cells (DCs) in order (81).

#### 3.2.1 M2-TAMs

Macrophages within the TME are designated as tumor-associated macrophages (TAMs). TAMs are the fundamental components within the TME, which perform a crucial function in the immunity remodeling of TME and affect response to immunotherapy (82, 83). A recent single-cell RNA sequencing revealed that the temporal and spatial distribution of macrophages was diverse in the TME of NSCLC and assisted tumor immune escape by initiating regulatory T-cell response (84). There are two classically polarized phenotypes of macrophages, including M1 (immune-activated type) and M2 (immunosuppressive type), and the latter is the main type of TAMs (72). TME enriched with more M2 macrophages is significantly associated with a worse response rate and prognosis for NSCLC patients receiving immunotherapy (85). However, TAMs can be re-engineered into M1-type to increase the response to ICIs treatment (86). In NSCLC, TAMs could promote tumor cell glycolysis by TNFα secretion and facilitate tumor hypoxia by increasing AMPK and PGC-1α, leading to decreased PD-L1 of tumor cells and T-cell infiltration in TME to cause immunotherapy resistance (87). Surprisingly, NSCLC patients enriched with PD-L1^+^ TAM in TME represented better survival for receiving PD-1/PD-L1 blockers (88). Additionally, another study suggested that PD-L1 mainly plays an effect in forming an immunosuppressive M2-type TAM (89). However, M2-type TAM could be reprogrammed into an immune-activated type by anti-PD-L1 treatment but not anti-PD-1 (89). Many genes or signaling pathways are involved in targeting TAM recruitment, activation, and survival, which can be applied as a targeting strategy to improve tumor immunotherapy (90, 91).

#### 3.2.2 NK cells

NK cells are powerful innate immune cells. NK cells perform a direct tumor-killing effect and indirectly enhance antitumor immunity mediated by T cells. Additionally, NK cells regulate DCs, macrophages, and neutrophils through cytotoxicity and cytokine release (92). Moreover, tumor-infiltrating NK cells could trigger T-cell-mediated immunity by stimulating the recruitment of DCs into TME, conferring improved tumor immune control (93). The infiltration of NK and plasma cells has been defined as a distinct immune subset in NSCLC, contributing to the most favorable prognosis (94). The high infiltration of NK cells in tumor tissue has been confirmed as a biomarker for predicting durable response to ICIs immunotherapy in NSCLC patients (95, 96). It is worth noting that there is a negative relationship between the density of TAMs and NK- and T-cell antitumor activities in NSCLC (97, 98).

#### 3.2.3 B cells

Tumor-infiltrating B cells have been identified as the most differential gene between immunotherapy responders and non-responders in patients with melanoma (99). Tertiary lymphoid structures (TLS) were also used as a marker of efficient immunotherapies due to initiating and/or maintaining local and systemic T- and B-cell mediated antitumor activity (100). B cells and plasma cells were found to co-present in TLS, and the abundance of intra-tumoral B cells was also linked to the prediction in the response efficacy of anti-PD-L1 in NSCLC (101, 102). B cells have also been found to exert antigen-presenting function to CD4^+^ TILs in TME to influence prognosis in NSCLC immunotherapy (102).

#### 3.2.4 CD4^+^ and CD8^+^ T cells

The presence of CD4^+^ T cells and CD8^+^ T cells in TME was associated with the objective clinical responses to anti-PD-1/PD-L1 blockade in NSCLC (103–107). And the predictive potency of CD4^+^ T cells and CD8^+^ T cells was more prominent in the PD-L1 positive sub-population. PD-1^+^ CD4^+^ T-cell was a negative predictor for immunotherapy availability in advanced NSCLC patients, while PD-1^+^CD8^+^ T-cell was a positive predictor (85, 103–105, 108). To complicate matters, multiple subsets or activity states of CD4^+^ T cells and CD8^+^ T cells have diverse effects on the response to immunotherapy. A single-cell sequencing analyzing T cell composition in NSCLC suggested that patients enriched with “pre-exhausted” CD8^+^ T cells (CD8-C4-GZMK), non-activated Tregs, and activated CD4^+^ cells had a much better prognosis than that in patients enriched with exhausted T cells (CD8-C6-LAYN and CD4-C7-CXCL13) and activated Tregs (109). Epigenetic changes-mediated high exhaustion of T cells is an important resistant mechanism to ICIs treatment (91, 110, 111), and therefore the “pre-exhausted” T cells might be alternative target for improving immunotherapy (109). Another study explored that NSCLC cells-derived exosomal circUSP7 could induce CD8^+^ T cell dysfunction to confer anti-PD-1 resistance (112). Recently, a phase I clinical trial confirmed the safety and feasibility of PD-1-edited T cells in NSCLC (113). Chimeric antigen receptor (CAR)-T cells, as another modified-T cell therapy, has attracted more and more interest in clinical applications as antitumor therapy for various solid tumors, including NSCLC, in recent years (114, 115). In summary, accumulated evidence validated various gene expression disorders or mutations in NSCLC cells impair the activity, tumor-cell-killing function, proliferation, and infiltration of CD8^+^ T-cells in TME, which contribute to exhausted CD8^+^ T-cells for immunotherapy resistance (91, 97, 111, 116–118).

## 4 Current clinical strategies for NSCLC after immunotherapy resistance

**Figure 5:**
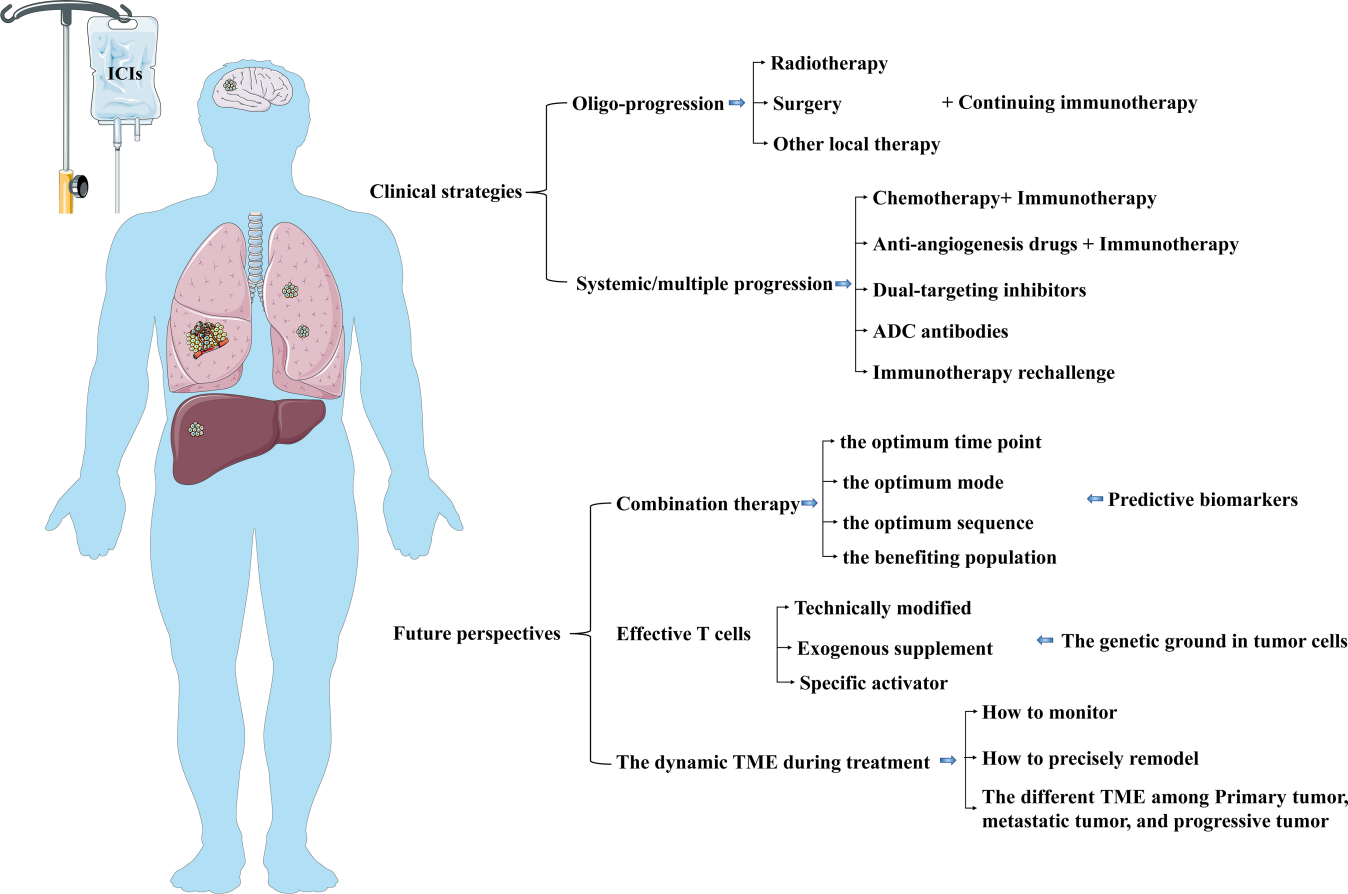
The current clinical strategies and future perspectives in NSCLC patients after immunotherapy resistance. The selection of clinical treatment for NSCLC progression after immunotherapy resistance should be dependent on the progressive mode. Further studies can pay attention to optimizing the combined therapy and exploring strategies to modulate infiltrating immune cells and timely reshape TME.

The progressive mode in NSCLC patients with immunotherapy resistance can be summarized into oligo-progression and systemic/multiple progression. The failure pattern of oligo-progression occurrence in 20% of NSCLC patients under treatment with PD-1/PD-L1 inhibitors (119). The oligo-progressive lesions are primarily involved in the brain, lung, and lymph nodes for immunotherapy-treated NSCLC patients (119). Clinical treatment selection for NSCLC progression after immunotherapy resistance should depend on the progressive mode (**Figure 5**).

### 4.1 Oligo-progression

The general cognition of the term “oligo-progression” is that the progressive metastases are up to 3–5 lesions and limited to 1–3 organs in the premise of a well-controlled metastatic disease (120, 121). Retrospective studies suggested that radiotherapy and/or surgery treating oligo-progressive sites combined with continuing immunotherapy could improve regional control rates and overall survival benefits (122–126). Many NSCLC patients receiving immunotherapy show primary resistance. Local radiation therapy on oligo-metastases improves immunotherapy response for NSCLC patients without multiple progression after primary systemic therapy. Radiotherapy might improve the response to immunotherapy in patients treated with ICI through its immunostimulatory effects and eradicating metastatic deposits (127). Radiation-killing tumor cells promote releasing and presenting tumor antigens, up-regulating PD-L1 expression on tumor cells, increasing TME infiltration of CD8^+^ T cells, and enhancing T cell-mediated immune response, contributing to improved antitumor immune response (128–130). The PEMBRO-RT trial(a multicenter, randomized phase 2) and MDACC (phase 1/2) trial found that NSCLC with PD-L1-negative tumors could obtain more survival benefits from the addition of radiotherapy than that in patients receiving ICI without radiotherapy (131, 132). For advanced NSCLC patients with oligo-progression or oligo-metastases, continuing immunotherapy combined with local treatment can eliminate primary and acquired resistance to ICIs, improving local control rate(LCR) and overall survival. Except for LCR, the improved overall survival from this combination is due to the distant antitumor effect on metastatic sites out of the radiation field by the radiotherapy-mediated immunoregulator effect. However, there are still many issues to be explored, such as the optimum patient selection, the radiotherapy scheme or fractionated dose selection, the number and location selection of radiation treating metastases, the selection of specific checkpoint inhibitors, and the sequence of radiotherapy combined with immunotherapy. There are many ongoing prospective trials about the effect of radiotherapy combined with immunotherapy in NSCLC patients with limited metastases or who oligo-progressed on ICI treatment or and we are looking forward to awaiting the outcome (127, 133, 134).

### 4.2 Systemic/multiple progression

#### 4.2.1 Combined therapy

Systemic combination therapies were currently clinically possible strategies for systemic/multiple progression in NSCLC treated with ICIs. BTCRC-LUN15-029(phase 2) suggested that NSCLC patients who progressed after ICIs alone or in combination with chemotherapy could benefit from pembrolizumab plus next-line chemotherapy (135). However, further research is needed to confirm the certain population that can benefit from continued immunotherapy after immunotherapy resistance. Although EGFR/ALK mutation was involved in the mechanism for ICI resistance, EGFR/ALK TKI combination with ICI usually served as a strategy for treating NSCLC patients with acquired EGFR-TKI resistance, not for patients with ICI resistance. However, limited clinical efficacy and a high incidence of treatment-related toxicities did not encourage the further application of this combination. Many clinical trials, such as CheckMate 370 (136), KEYNOTE-021 (137), GEFTREM (138), LUX-Lung-IO (139), have shown that EGFR or ALK inhibitors combined with ICIs were feasible but limited efficacy and more toxicity in treating NSCLC patients with newly diagnosed or refractory or PD after first-line therapy. Furthermore, the growing evidence supported the combination of anti-angiogenic agents and ICIs (140, 141). Ramucirumab combined with pembrolizumab showed an encouraging antitumor activity with acceptable toxicities in NSCLC patients, having progressed on one to three lines of previous therapy (142). A pooled analysis from 23 prospective studies confirmed that ICIs combined with anti-angiogenic agents with favorable antitumor activity and manageable toxic effects might be a new option for NSCLC patients, especially those who received no treatment or chemotherapy intolerance (143). However, especially for treating NSCLC patients with PD after ICI-pretreated, the application of this combination therapy is in the exploring phase. More and more prospective trials have been conducted to identify the efficiency and safety of PD-1/L1 inhibitors plus anti-angiogenesis drugs(Cabozantinib, Sitravatinib, Lenvatinib, Nintedanib) in NSCLC patients previously treated with an ICI (144–150). Cabozantinib is a multi-targeted tyrosine kinase inhibitor (TKI) and reshapes an immune-active TME by the inhibition of MET and TAM receptor kinases (TYRO3, AXL, MER) (151, 152). Sitravatinib is another multi-targeted TKI that inhibits VEGFR, TAM receptors (TYRO3, AXL, MERTK), and Split family receptors, which reduce the proliferation of immunosuppressive cells, initiate T cell infiltration into TME, decrease T cell exhaustion, promote M1 macrophage polarization (153, 154). The anti-VEGFR drug Lenvatinib was found to perform immunoregulation by reducing TAMs infiltration and increasing the activity of CD8^+^ T cells (155, 156). Another anti-angiogenic agent Nintedanib also plays an antitumor immunity by remodeling TME through increased tumoral infiltration of CD8^+^ T cells and granzyme B production (157). Anti-PD-1/PD-L1 and other non-PD-1/PD-L1 blockers are alternative therapeutic strategies for NSCLC patients with immunotherapy resistance. However, Durvalumab plus tremelimumab(anti-CTLA-4) had minimal benefits in NSCLC patients with progression after anti-PD-1 therapy (158). Other trials about combining with Vibostolimab(anti-TIGIT) (NCT02964013, NCT04725188) have not been completed. Moreover, TME modulators, such as MDM2, SEMA4D, and HDAC inhibitors, combined with ICIs were explored to apply in NSCLC patients with refractory and failure after immunotherapy, resulting in safety but limited benefits (159–162). These preliminary results need to be validated in further studies, including a larger sample, adjusted scheme, or changed time point of combined treatment. There are other ongoing clinical trials to investigate the practicability of new drugs such as AK112(dual-targeting PD-1/VEGF) or KN046(dual-targeting PD-1/CTLA-4), or SAR408701(anti-CEACAM5) in antagonizing immunotherapy resistance (163–165). Increasing clinical trials are ongoing to investigate and explore the safety and efficacy of a new combination treatment or drug in NSCLC patients with ICIs resistance (**Table 1**). We expect these trials’ results will provide additional therapeutic options for ICI-resistant NSCLC in the short term.

**Table 1:** Clinical trials investigating new treatments to overcome ICI resistance in NSCLC.

Intervention/treatment	Drug	ClinicalTrials.gov Identifier	Phase	Patients	Recruitment Status
**Vaccines**					
	RO7198457	NCT03289962	I	ICI-naiıve and pretreated	Active, not recruiting
	Viagenpumatucel-L	NCT02439450	I/II	Including ICI-pretreated	Completed
	Autologous LN-145	NCT04614103	II	Documented PD after ICI-pretreated	Recruiting
	STEMVAC	NCT05242965	II	ICI-pretreated	Recruiting
**Adoptive cell therapy**					
	Letetresgene Autoleucel T-cells	NCT03709706	I/II	Including ICI-pretreated	Terminated
**Targeting-drive gene**					
	Cabozantinib (c-MET)	NCT03600701	II	ICI-naive and pretreated	Recruiting
	Cobimetinib(MEK)	NCT03170960	I/II	ICI-naive and pretreated	Active, not recruiting
	SAR408701(anti-CEACAM5)	NCT04394624	II	Having PD after ICI/chemotherapy-pretreated	Recruiting
**Anti-angiogenic agents**					
	Cabozantinib	NCT04471428	III	Documented PD after previous ICI treatment	Active, not recruiting
	Sitravatinib	NCT03906071	III	Prior treatment with ICI and chemotherapy	Active, not recruiting
	Lenvatinib	NCT03976375	III	Documented PD after previous ICI treatment	Active, not recruiting
	Nintedanib	NCT03377023	I/II	ICI-naive and pretreated with chemotherapy, ICI, or targeted therapy	Active, not recruiting
	Ramucirumab	NCT03689855	II	ICI-pretreated	Active, not recruiting
		NCT04340882	II	Having PD after ICI+chemotherapy	Recruiting
**TME modulators**					
	Bintrafusp Alfa(anti- TGF-β)	NCT04396535	II	ICI+chemotherapy-pretreated	Active, not recruiting
	Entinostat (anti-HDAC)	NCT01928576	II	ICI-naiıve and pretreated	Active, not recruiting
	Mocetinostat(anti-HDAC)	NCT02954991	II	ICI-pretreated	Completed
	Vibostolimab (anti-TIGIT)	NCT04725188	II	Having PD after Chemotherapy and ICI	Active, not recruiting
	Abemaciclib(anti-CDK4/6)	NCT02779751	I	ICI-naive and pretreated	Active, not recruiting

#### 4.2.2 Immunotherapy rechallenge

Should NSCLC patients with prior anti-PD-1/PD-L1 therapy with a durable response be given a second course of ICI if their disease progresses? For PD-L^+^ NSCLC patients who had progression after 35 cycles/2 years of pembrolizumab, pembrolizumab retreatment showed that the disease control rate was over 77% (166). Another real-world setting also found evidence supporting that nivolumab retreatment could bring better responses in NSCLC patients who had a long-term response to first-course treatment (167). In addition, other reports also found that only limited patients could benefit from immunotherapy retreatment (168, 169), so it is necessary to explore effective predictive biomarkers to screen the optimum population.

## 5 Conclusion and future perspectives

Although immunotherapy improves survival prognosis in NSCLC patients, primary and acquired resistance impair long-term clinical benefits in the partial population. There is a negative relationship between the ORR of immunotherapy and the occurrence of acquired resistance. 74% of NSCLC patients with an effective initial response to immunotherapy will experience disease progression within 5 years. The mechanism of immunotherapy resistance is complex, dynamic, and interdependent, mainly involving intrinsic factors(tumor cells) and extrinsic factors (TME infiltrating immune cells). In the clinical appliance, therapeutic strategies for NSCLC progression after immunotherapy resistance is still in the exploratory stage. The progressive mode after immunotherapy resistance should be taken into consideration for further treatment. Radiotherapy, chemotherapy, anti-angiogenesis drugs, and TME modulators can synergistically enhance immunotherapy by regulating the process of the cancer-immunity cycle, including tumor antigen release, presentation, and TME infiltrating immune cells. However, these synergistic effects are uncontrollable and unpredictable. In further studies, we still face many challenges, such as how to find the optimum time point, mode, sequence of combination therapy, and the population who can obtain more benefits from the combination therapy, how to find the corresponding predictive biomarkers, how to confirm the optimum treatment for NSCLC patients characterized by distinct or multiple genetic backgrounds. A prerequisite for a clinical response to immunotherapy is a normal cancer-immunity cycle, of which effective T cells are vital policymakers and executants for antitumor immunity. Therefore, technically modified-T cell therapy attains more and more attention. In addition, also worth considering are the effect of the genetic background of tumor cells on native or artificially reprogrammed immune cells and how to monitor and precisely remodel the dynamic TME during treatment (**Figure 5**).

## Author contributions

SZ: contribution to data collection, writing, and editing figures. HY: conception and design of the study, revising the manuscript critically. All authors contributed to the article and approved the submitted version.
